# Glycolysis-Stimulated Esrrb Lactylation Promotes the Self-Renewal and Extraembryonic Endoderm Stem Cell Differentiation of Embryonic Stem Cells

**DOI:** 10.3390/ijms25052692

**Published:** 2024-02-26

**Authors:** Qiman Dong, Qingye Zhang, Xiaoqiong Yang, Shanshan Nai, Xiaoling Du, Lingyi Chen

**Affiliations:** Institute of Translational Medicine, Tianjin Union Medical Center, State Key Laboratory of Medicinal Chemical Biology, Tianjin Key Laboratory of Protein Sciences, Frontiers Science Center for Cell Responses, National Demonstration Center for Experimental Biology Education and College of Life Sciences, Nankai University, Tianjin 300074, China; 18330225980@163.com (Q.D.); 17695649501@163.com (Q.Z.); 15516622620@163.com (X.Y.); 15701207153@163.com (S.N.); duxiaoling@nankai.edu.cn (X.D.)

**Keywords:** lactylation, Esrrb, self-renewal, XEN differentiation, embryonic stem cells

## Abstract

Embryonic stem cells (ESCs) favor glycolysis over oxidative phosphorylation for energy production, and glycolytic metabolism is critical for pluripotency establishment, maintenance, and exit. However, an understanding of how glycolysis regulates the self-renewal and differentiation of ESCs remains elusive. Here, we demonstrated that protein lactylation, regulated by intracellular lactate, contributes to the self-renewal of ESCs. We further showed that Esrrb, an orphan nuclear receptor involved in pluripotency maintenance and extraembryonic endoderm stem cell (XEN) differentiation, is lactylated on K228 and K232. The lactylation of Esrrb enhances its activity in promoting ESC self-renewal in the absence of the LIF and XEN differentiation of ESCs by increasing its binding at target genes. Our studies reveal the importance of protein lactylation in the self-renewal and XEN differentiation of ESCs, and the underlying mechanism of glycolytic metabolism regulating cell fate choice.

## 1. Introduction

Embryonic stem cells (ESCs), derived from the inner cell mass (ICM) of preimplantation blastocysts, hold great potential in biomedical research and regenerative medicine due to their ability to self-renew indefinitely and differentiate into all cell types in the body [1,2]. Enhanced glycolysis is a unique metabolic feature of ESCs. Regardless of the availability of oxygen, ESCs produce energy preferentially through glycolysis, in which glucose is converted to pyruvate and lactate. In contrast, differentiated cells bias energy production through oxidative phosphorylation (OXPHOS), in which pyruvate is converted into acetyl-CoA, followed by the tricarboxylic acid cycle (TCA cycle) [3,4,5,6,7]. Moreover, the switch between glycolysis and OXPHOS plays a critical role in regulating the self-renewal and differentiation of ESCs, as well as somatic cell reprogramming. The inhibition of glycolysis compromises the self-renewal of naïve human ESCs and feeder-free primed human ESCs [8]. The blockade of glycolytic enzyme activity reduces reprogramming efficiency by Yamanaka factors [9]. During the early differentiation phase, glycolytic inhibitors upstream of acetyl-CoA cause the differentiation of pluripotent stem cells (PSCs), implying that acetyl-CoA produced by glycolysis suppresses PSC differentiation [10].

Even though the functions of glycolytic metabolism in pluripotency establishment, maintenance, and exit, have been demonstrated, how glycolysis regulates these events remains largely unknown. It has been shown that the accumulation of lactate, which is a metabolic product of glycolysis and derived from pyruvate by lactate dehydrogenase (Ldh), sustains the self-renewal of ESCs even in differentiation conditions, and promotes the differentiation of extraembryonic endoderm stem (XEN) cells in vitro [11,12]. Lactate is also a substrate of lactylation. Histone lysine lactylation (Kla) regulates gene expression in M1 macrophage polarization, the pro-inflammatory activation of microglia, immune homeostasis post–myocardial infarction, ocular melanoma, and somatic cell reprogramming [13,14,15,16,17]. In addition, the Kla of non-histone proteins, such as METTL3 and the ubiquitin–proteasome system, modulates the function of tumor-infiltrating myeloid cells and systemic lupus erythematosus pathogenesis, respectively [18,19]. Therefore, we speculated that glycolysis might promote pluripotency maintenance through the lactate and Kla of proteins.

Estrogen-related receptor beta (Esrrb) is a well-known transcription factor involved in the maintenance and establishment of pluripotency [20,21,22,23,24]. Esrrb overexpression is sufficient to maintain ESC self-renewal in the absence of LIF [20,21]. Esrrb, together with Oct4 and Sox2, are able to reprogram mouse embryonic fibroblasts (MEFs) to induced pluripotent stem cells (iPSCs) [23]. With a combination of five transcription factors, Gata3, Eomes, Tfap2c, Myc, and Esrrb, fibroblasts can be reprogrammed into iPSCs, induced trophoblast stem cells, and induced XEN cells, concomitantly. Esrrb and Eomes drive the XEN and the trophectodermal fates, respectively [25]. Consistently, Esrrb promotes XEN cell differentiation from ESCs by binding to poised enhancers of XEN genes [26].

In this study, we first validated that the inhibition of glycolysis by the Ldh inhibitor sodium oxamate (SO) or through the knockout (KO) of *Ldha* compromises the self-renewal of ESCs. Analyzing the lactylome profiles in ESCs with and without SO treatment (manuscript under review in *iScience*), we focused on estrogen-related receptor beta (Esrrb), lactylation, which might regulate the self-renewal of ESCs. The lactylation sites on Esrrb were mapped to K228 and K232. Esrrb lactylation mimic mutant KQ (K228Q and K232Q) is more potent than WT Esrrb and unlactylated mimic mutant KR (K228R and K232R) in activating pluripotency genes and maintaining the self-renewal of ESCs. In addition, during ESC differentiation to XEN cells, the lactylation of Esrrb promotes its binding to XEN lineage genes and, consequently, the XEN differentiation of ESCs. In summary, our data provide mechanistic insights on how enhanced glycolysis contributes to ESC self-renewal and XEN differentiation, and reveal that the glycolytic metabolism facilitates the self-renewal of ESCs and XEN differentiation through the lactylation of Esrrb.

## 2. Results

### 2.1. Ldh Inhibition Compromises the Self-Renewal of ESCs

**Figure 1:**
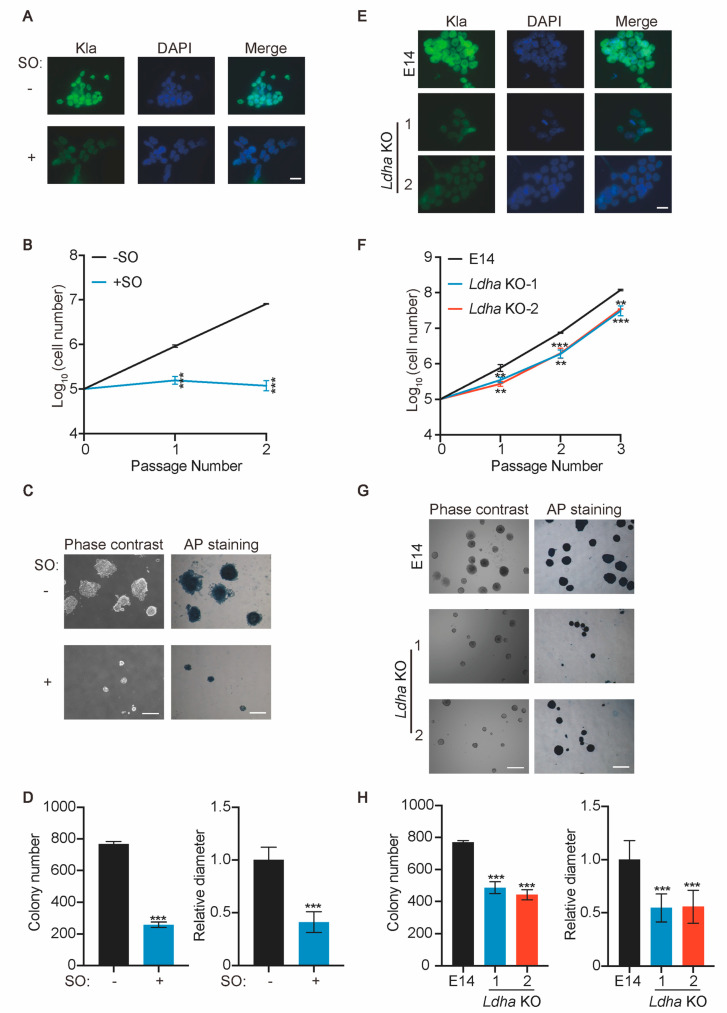
Inhibition of Ldh compromises self−renewal of ESCs. (**A**) Immunofluorescence showing global expression levels of Kla after E14 treatment with or without 20 mM SO for 24 h. Scale bar: 20 μm. (**B**) Growth curves of ESCs cultured with and without 20 mM SO. (**C**) Colony−forming assay of E14 treated with and without SO. Representative images of colony−forming assay and AP staining are shown. Scale bar: 500 μm. (**D**) Number and diameter of control and SO−treated ESC colonies described in (**C**). (**E**) Immunofluorescence showing global expression levels of Kla following loss of *Ldha*. Two independent *Ldha* KO ESC lines, marked as 1 and 2, were subjected to an immunofluorescence assay. Scale bar: 20 μm. (**F**) Growth curves of E14 and *Ldha* KO ESCs. (**G**) Colony−forming assay of E14 and *Ldha* KO ESCs. Representative images of colony−forming assay and AP staining are shown. Scale bar: 500 μm. (**H**) Number and diameter of E14 and *Ldha* KO ESC colonies described in (**G**). For growth curves and colony formation, n = 3. Data are presented as average ± SD. Statistical analysis was performed with an unpaired two−tailed Student’s *t* test. ** *p* < 0.01; *** *p* < 0.001.

To validate the importance of lactate and Kla in pluripotency maintenance, we employed a pharmacological inhibitor of Ldh, sodium oxamate (SO), to prevent the conversion of pyruvate to lactate and reduce Kla levels. The SO treatment of WT E14 ESCs decreases the Kla level (Figure 1A). The inhibition of Ldh by SO reduces the proliferation rate of ESCs (Figure 1B). The colony-forming capacity of ESCs is impaired by SO treatment, indicated by a reduced colony number and smaller colony size (Figure 1C,D). However, the expression of alkaline phosphatase (AP) and pluripotency genes, including Nanog and Oct4, is not affected by SO treatment (Figure 1B and Figure S1A,B).

To rule out the off-target effect of SO, we also tried to suppress Kla by knocking out Ldh. Given the low expression level of *Ldhc* and *Ldhd* in mouse ESCs (manuscript under review in *iScience*), only the effects of *Ldha* and *Ldhb* KO on ESC self-renewal were analyzed. The KO of *Ldha* reduces the level of Kla, the proliferation rate, and the colony-forming capacity of ESCs, but does not affect the expression of pluripotency genes (Figure 1E–H and Figure S1C,D), similar to the phenotypes caused by SO treatment. In contrast, the KO of *Ldhb* does not alter the level of Kla, or lead to any defects in proliferation and colony formation (Figure S2A–C). Given the abundance of *Ldha* in ESCs, it is expected that *Ldha* KO ESCs would display more severe phenotypes than *Ldhb* KO ESCs.

Taken together, these data suggest that the inhibition of Ldh suppresses protein lactylation and impairs the self-renewal of ESCs.

### 2.2. Esrrb Is Lactylated on K228 and K232

**Figure 2:**
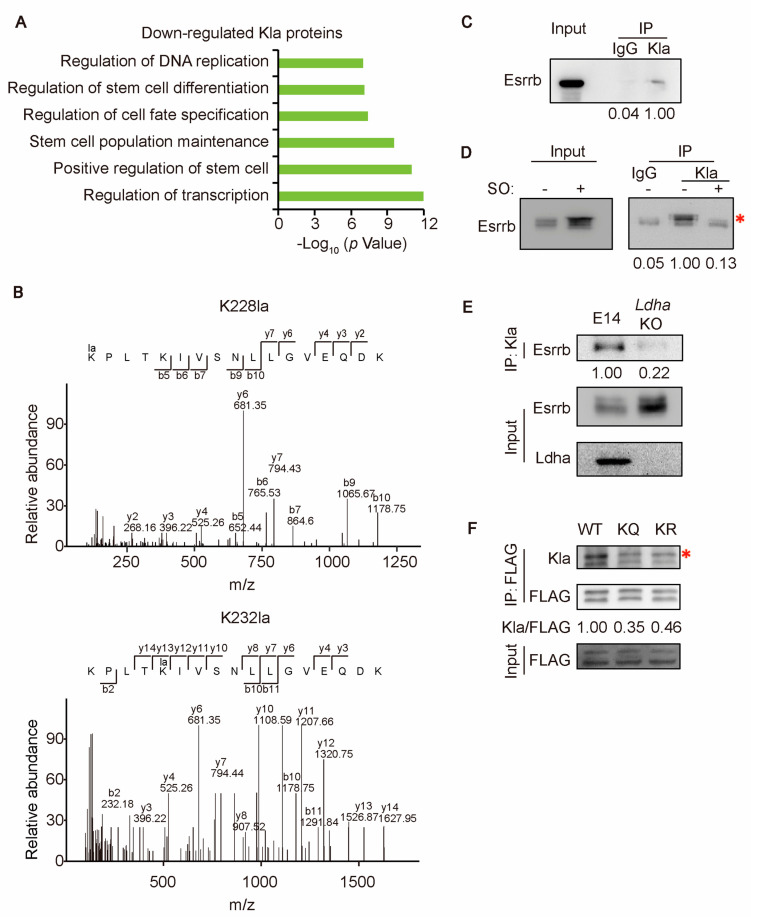
Esrrb is lactylated on K228 and K232. (**A**) GO analysis of down−regulated Kla proteins upon SO treatment. The procedure and result of lactylome analysis are described elsewhere (manuscript under review in *iScience*). (**B**) MS/MS spectra showing 2 lactylated sites (K228 and K232) of Esrrb. The b and y ions refer to the N−terminal and C−terminal parts of the peptide, respectively. (**C**) After IP with IgG or anti−Kla antibody, using E14 ESC lysate, Esrrb was detected by Western blot. (**D**) E14 ESCs with and without SO treatment were subjected to IP with IgG or anti−Kla antibody, and Western blot was performed to detect Esrrb. (**E**) After Anti−Kla IP with E14 and *Ldha* KO ESC lysates, Esrrb and Ldha were detected by Western blot. (**F**) E14 ESCs stably expressing FLAG−tagged WT, KQ, and KR Esrrb were subjected to anti−FLAG IP. Western blot was carried out to detect Kla and FLAG in the IP samples. Red asterisks mark the specific band of Esrrb. Bands of interest were quantified using Image J 1.51 software, and the results are shown below the corresponding images.

To investigate how protein lactylation stimulated by glycolysis regulates the self-renewal and differentiation of ESCs, we analyzed the lactylome profiles of ESCs with and without SO treatment (manuscript under review in *iScience*, PXD050051). Gene Ontology (GO) analysis revealed that down-regulated Kla proteins are involved in various pluripotency-associated biological processes, including the positive regulation of stem cells, stem cell population maintenance, and the regulation of stem cell differentiation (Figure 2A). Among the down-regulated Kla proteins associated with stem cell maintenance and the regulation of transcription, Esrrb, a pluripotency transcription factor involved in the maintenance and establishment of pluripotency, as well as XEN cell differentiation [20,21,22,23,24,26], drew our attention. We hypothesized that Kla might play a role in modulating the activity of Esrrb, hence regulating the self-renewal and differentiation of ESCs.

The lactylome analysis revealed that upon SO treatment, the levels of Esrrb K228 and K232 lactylation (K228la and K232la) were decreased by 0.672- and 0.616-fold, respectively (Figure 2B) (manuscript under review in *iScience*). To validate the lactylome data, immunoprecipitation (IP) assays were performed. IP with anti-Kla antibodies was able to pull down endogenous Esrrb (Figure 2C), indicating that Esrrb is lactylated. The inhibition of Ldh, either by the SO or by KO of *Ldha*, reduces the lactylation level of Esrrb (Figure 2D,E), suggesting that the lactylation of Esrrb is stimulated by glycolysis. To map the lactylation sites on Esrrb, the K228 and K232 of Esrrb were simultaneously mutated to glutamine (Q) or arginine (R) to mimic lactylated and unlactylated statuses, respectively. The resulting Esrrb mutants were named KQ and KR mutants. The Kla levels of both KQ and KR mutants are lower than that of WT Esrrb (Figure 2F), confirming that K228 and K232 are major lactylation sites on Esrrb.

### 2.3. Esrrb KQ Mutant Is More Potent in Pluripotency Maintenance

**Figure 3:**
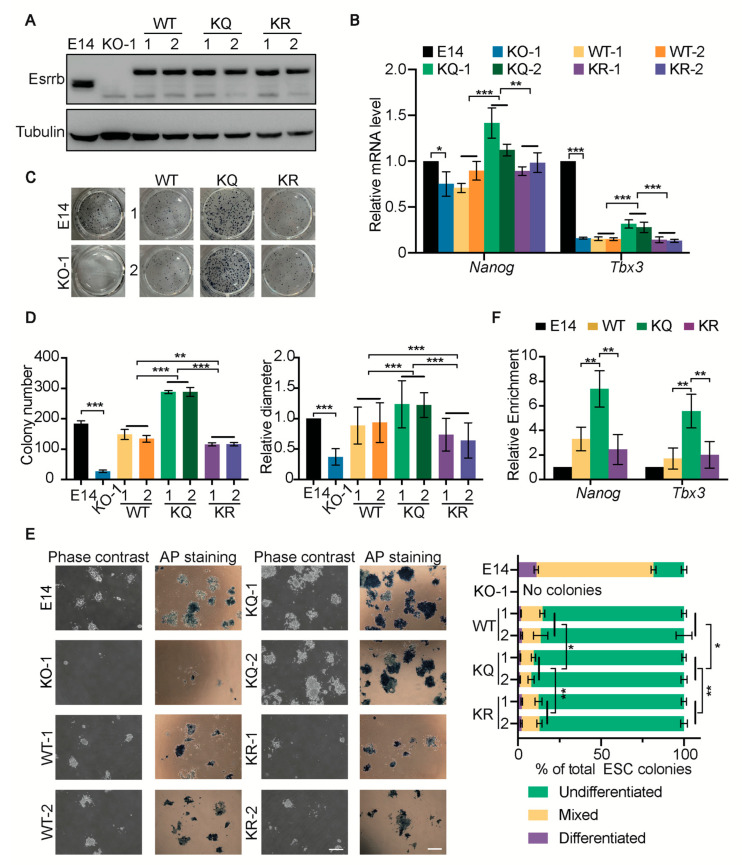
Lactylated Esrrb is more potent in maintaining ESC self−renewal. (**A**) *Esrrb* KO−1 ESC lines stably expressing FLAG−tagged WT, KQ, and KR Esrrb were established and named WT, KQ, and KR ESCs. Western blot was performed to show the expression levels of Esrrb in these cell lines. Two clones of WT, KQ, and KR ESCs were included and are marked as 1 and 2. (**B**) The expression of *Nanog* and *Tbx3* RNA in E14, *Esrrb* KO−1, WT, KQ, and KR ESCs assessed by qRT−PCR. (**C**) Colony−forming assay of E14, *Esrrb* KO−1, WT, KQ, and KR ESCs in the absence of LIF. (**D**) The number and diameter of ESC colonies described in (**C**). (**E**) The bright-field images of colony−forming assays (**C**), before and after AP staining, are shown on the left. Colonies were scored according to AP staining, and categorized as undifferentiated (>85% AP−positive), mixed (15–85% AP−positive), or differentiated (<15% AP−positive). The right panel shows the fractions of undifferentiated, mixed, and differentiated colonies in each cell line. (**F**) ChIP−qPCR of Esrrb binding at the *Nanog* and *Tbx3* loci. ChIP assays were performed in E14, WT, KQ, and KR ESCs using anti−FLAG antibodies. Three independent experiments were analyzed, and data are presented as average ± SD. Statistical analysis was performed with two−way ANOVA for (**B**,**D**,**E**) and one−way ANOVA for (**F**). * *p* < 0.05; ** *p* < 0.01; *** *p* < 0.001. Scale bar: 500 μm.

It has been demonstrated that the KO of *Esrrb* results in impaired pluripotency, such as the decreased expression of pluripotency markers, a reduced proliferation rate, and weaker AP positivity [20,21,22]. To elucidate the function of Esrrb lactylation, we first constructed *Esrrb* KO ESCs using CRISPR/Cas9 (Figure S3A). Two independent clones (*Esrrb* KO-1 and *Esrrb* KO-2) were confirmed to be Esrrb-null ESCs by DNA sequencing and Western blot (Figure S3B–D). The abovementioned *Esrrb* KO phenotypes were observed in our *Esrrb* KO ESC lines (Figure S3E–H). Next, *Esrrb* KO-1 ESC lines expressing FLAG-tagged WT, KQ, and KR Esrrb, denoted as WT, KQ, and KR ESCs, were established (Figure 3A). In the presence of leukemia-inhibitory factor (LIF), WT, KQ, and KR Esrrb rescued the phenotypes of *Esrrb* KO, including reduced colony-forming capacity, differentiated colony morphology, and decreased proliferation rate. Yet, no difference in WT, KQ, and KR Esrrb in rescuing these *Esrrb* KO phenotypes was detected (Figure S3E–H). Nevertheless, we found that only KQ Esrrb partially rescues the expression *Nanog* and *Tbx3* RNA, while WT and KR Esrrb have no obvious effect on *Nanog* and *Tbx3* expression (Figure 3B), implying that the lactylation of Esrrb might have functional effect.

To better demonstrate the functional effect of Esrrb lactylation, we compared the functions of WT, KQ, and KR Esrrb in maintaining the undifferentiated state of ESCs in the absence of LIF. Under the LIF withdrawal condition, KQ ESCs form more colonies than WT and KR ESCs (Figure 3C,D). Moreover, AP staining revealed that a larger fraction of KQ colonies is undifferentiated compared with WT and KR colonies (Figure 3E). These data suggest that KQ Esrrb is more potent in maintaining the self-renewal of ESCs in the absence of LIF.

Furthermore, ChIP assays reveal that KQ Esrrb is more enriched at the *Nanog* and *Tbx3* loci than WT and KR Esrrb (Figure 3F), suggesting that Esrrb lactylation might enhance its DNA binding in pluripotency genes to promote ESC self-renewal.

### 2.4. Esrrb Lactylation Promotes XEN Differentiation

It has been shown that *Esrrb* KO ESCs fail to differentiate into XEN cells (Figure S4A–C) [26]. Interestingly, intracellular lactate enhances ESC differentiation toward XEN cells in vitro [12]. Hence, we further explored whether Esrrb lactylation also plays a role in the XEN differentiation of ESCs.

**Figure 4:**
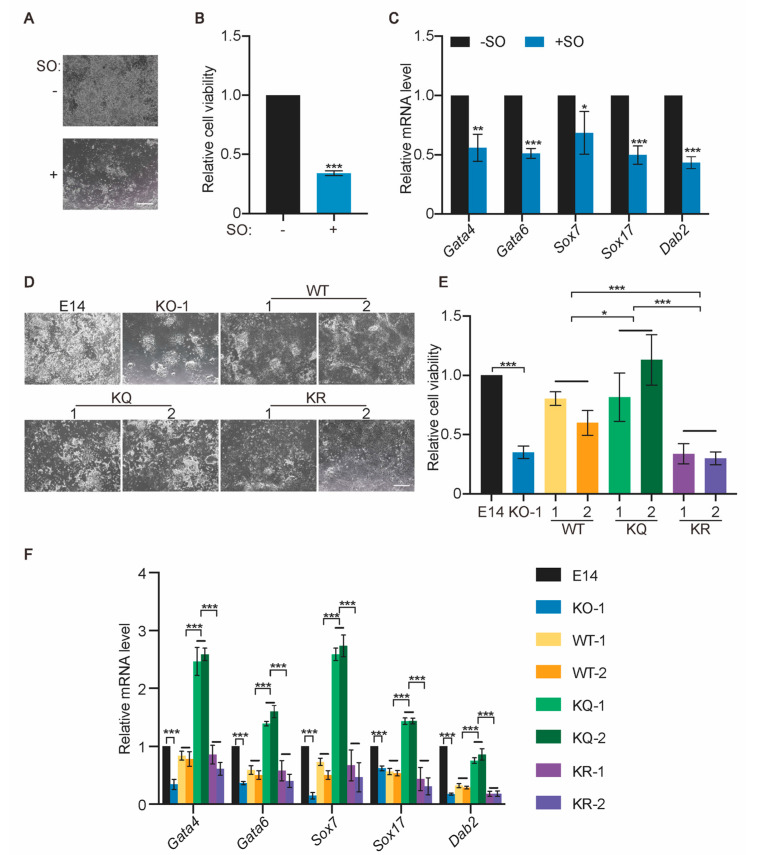
Lactylation of Esrrb promotes XEN differentiation of ESCs. (**A**) E14 ESCs were differentiated toward the XEN lineage in the presence and absence of 1 mM SO for 8 days. Representative phase-contrast images of XEN cells with and without SO are shown. (**B**) The relative cell viability of XEN cells described in (**A**). (**C**) The expression of selected XEN genes in cells described in (**A**). (**D**) The representative phase−contrast images of XEN cells differentiated from E14, *Esrrb* KO−1, WT, KQ, and KR ESCs. Two clones of WT, KQ, and KR ESCs were included and are marked as 1 and 2. (**E**) The relative cell viability of XEN cells described in (**D**). (**F**) The expression of selected XEN genes in XEN cells described in (**D**). Three independent experiments were analyzed, and data are presented as average ± SD. Statistical analyses were performed with unpaired two−tailed Student’s *t* test (**B**,**C**) and two−way ANOVA (**E**,**F**). * *p* < 0.05; ** *p* < 0.01; *** *p* < 0.001. Scale bar: 500 μm.

We first confirmed the importance of glycolysis and lactate in XEN differentiation. SO prevents ESC differentiation to XEN cells, indicated by reduced cell viability and lower expression levels of XEN genes, such as *Gata4*, *Gata6*, *Sox7*, *Sox17*, and *Dab2* (Figure 4A–C). Consistently, *Ldha* KO, but not *Ldhb* KO, compromises XEN differentiation (Figure S4D–I). These data suggest that the inhibition of lactate production, and subsequently, lactylation, prevents XEN differentiation.

We then addressed whether WT, KQ, and KR Esrrb have any functional difference in promoting XEN differentiation. WT-, KQ-, and KR-rescued *Esrrb* KO-1 ESC lines (WT, KQ, and KR ESCs), as well as E14 and *Esrrb* KO-1 ESCs, were induced to undergo XEN differentiation. KQ Esrrb is most potent in promoting ESC differentiation toward the XEN lineage. More KQ cells are viable after 8-day XEN differentiation, and the expression levels of XEN genes are higher in KQ cells than those in WT and KR cells (Figure 4D–F). These data imply that the lactylation of Esrrb promotes XEN differentiation of ESCs.

### 2.5. Lactate Regulates XEN Differentiation Mainly by Lactylating Esrrb 

**Figure 5:**
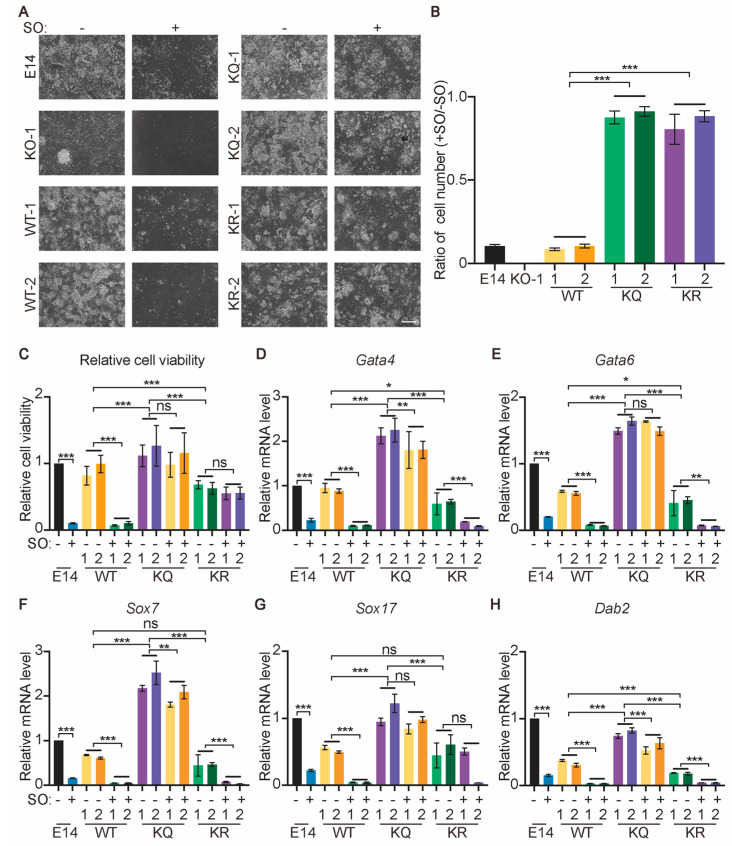
Lactate promotes XEN differentiation mainly by lactylating Esrrb. (**A**) Representative phase−contrast images of XEN cells differentiated from E14, *Esrrb* KO−1, WT, KQ, and KR ESCs with and without 1 mM SO. Two clones of WT, KQ, and KR ESCs were included and are marked as 1 and 2. Scale bar: 500 μm. (**B**,**C**) Cell numbers were counted for the cells described in (**A**). The ratio of XEN cell number with SO to XEN cell number without SO (**B**) and the relative cell viability (**C**) were calculated and plotted. (**D**−**H**) The expression of selected XEN genes in the cells described in (**A**). Three independent experiments were analyzed, and data are presented as average ± SD. Statistical analysis was performed with two−way ANOVA. * *p* < 0.05; ** *p* < 0.01; *** *p* < 0.001, ns: not significant.

Next, to elaborate the contribution of Esrrb lactylation to lactate promoting XEN differentiation, WT-, KQ-, and KR-rescued *Esrrb* KO-1 ESCs, as well as E14 and *Esrrb* KO-1 ESCs, were induced to undergo XEN differentiation in the presence and absence of SO. In terms of cell viability, KQ and KR ESCs are resistant to SO, while WT ESCs are sensitive to SO treatment (Figure 5A–C), suggesting that the lactylation of Esrrb K228 and K232 is the major event mediating the effect of lactate in stimulating XEN differentiation. However, the expression levels of some XEN genes, such as *Gata4*, *Sox7*, and *Dab2*, are reduced, even in KQ cells (Figure 5D–H). This implies that except for Esrrb K228la and K232la, lactate has additional mechanisms to regulate XEN differentiation.

### 2.6. Lactylation of Esrrb Enhances Its Binding to XEN Genes 

**Figure 6:**
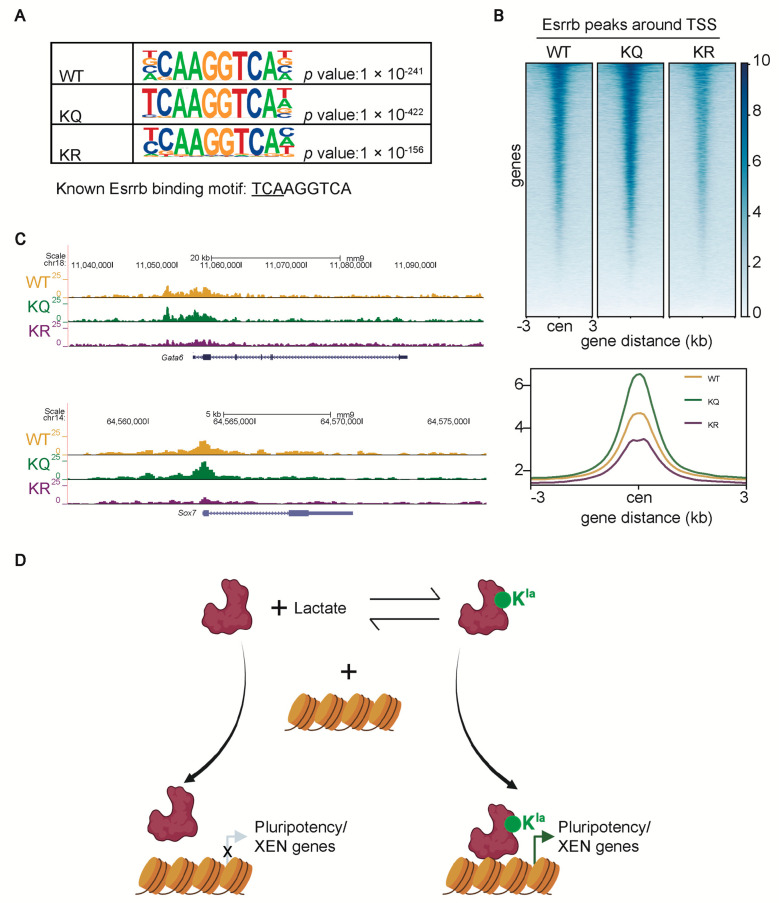
Lactylation of Esrrb enhances its binding to XEN genes. ChIP−seq assays were performed in WT, KQ, and KR differentiated ESCs toward the XEN lineage using anti-FLAG antibodies. (**A**) Motif analysis for WT, KQ, and KR Esrrb−binding sites identified by ChIP−seq. (**B**) Heatmap plots (upper panel) and averaged profiles of the WT, KQ, and KR Esrrb ChIP−seq signals at TSSs. (**C**) Integrative genomics viewer (IGV) tracks at XEN gene loci, *Gata6* and *Sox7*. (**D**) A working model for Esrrb lactylation to promote XEN differentiation.

To understand how lactylated Esrrb promotes XEN differentiation, we conducted chromatin immunoprecipitation followed by sequencing (ChIP-seq) in WT-1, KQ-1, and KR-1 cells after 8-day XEN differentiation using anti-FLAG antibodies. Regardless of the lactylation status, the binding motifs of WT, KQ, and KR Esrrb identified by our ChIP-seq analysis are identical to the known Esrrb-binding motif TCAAGGTCA (Figure 6A). Yet, it is notable that the binding motif of KQ Esrrb is more similar to the known Esrrb-binding motif, particularly in the underlined three nucleotides, which contribute to the binding affinity [27]. Consistently, we found that the binding of KQ Esrrb at transcription start sites (TSSs) is stronger than that of WT and KR Esrrb (Figure 6B). In addition, stronger binding of KQ Esrrb is also observed in XEN gene loci, such as *Gata6* and *Sox7* (Figure 6C). These data imply that the lactylation of Esrrb enhances its binding to XEN genes to promote XEN differentiation.

## 3. Discussion

It has been well recognized that ESCs, similar to cancer cells, preferentially utilize glycolysis, rather than OXPHOS, for energy supply, and that enhanced glycolysis is critical for the fast self-renewal of ESCs [4,5,6]. Enhanced glycolysis may facilitate fast proliferation by supplying sufficient anabolic intermediates and minimizing reactive oxygen species (ROS) production [7,28]. Moreover, it has been shown that during the initial stage of differentiation, acetyl-CoA produced by glycolysis may prevent PSC differentiation by inhibiting histone deacetylation, indicating the importance of the pyruvate–acetyl-CoA step in pluripotency maintenance [10]. In contrast, our studies reveal a pivotal role of the pyruvate–lactate step in pluripotency maintenance, as well as XEN differentiation. We demonstrated that lactate and protein lactylation derived from glycolysis are essential for pluripotency maintenance. Lactylome analysis revealed that many proteins involved in the self-renewal and differentiation of ESCs are lactylated. Moreover, we proved that the lactylation of Esrrb K228 and K232 enhances its capacity to promote ESC self-renewal and XEN differentiation. Mechanistically, the lactylation of Esrrb enhances its binding to pluripotency and XEN genes, thus facilitating self-renewal and XEN differentiation (Figure 6D).

Shown in Figure 5D–H, some XEN genes, such as *Gata4*, *Sox7*, and *Dab2*, are sensitive to SO treatment even in KQ cells, suggesting that in addition to Esrrb lactylation, other factors might contribute to lactate- and lactylation-regulated XEN differentiation. Consistent with this note, the functions of histone H3 lysine 18 lactylation (H3K18la) in somatic cell reprogramming and activating germline and cleavage embryo genes in ESCs have been demonstrated [16,29]. In addition, our lactylome analysis identified other lactylated proteins, such as Dgcr8 and Sall4, involved in ESC self-renewal and differentiation. Dgcr8 is an RNA-binding protein essential for microRNA processing. When induced to differentiation, *Dgcr8* KO ESCs fail to fully silence pluripotency genes and retain their ESC colony-forming capacity [30]. Sall4 is a transcription factor involved in the self-renewal of ESCs and XEN cells [31,32,33,34]. Thus, further investigation is required to comprehensively understand how the lactylation of proteins regulates the self-renewal and differentiation of ESCs.

The lactylation of mitochondrial proteins, such as PDHA1 and CPT2, reduces OXPHOS activity under hypoxic conditions [35], suggesting that protein lactylation may regulate cellular metabolism. Given the importance of cellular metabolism in cell fate regulation, it is worth investigating whether and how the metabolic profile of ESCs, particularly the glycolytic and mitochondrial respiratory fluxes, changes upon Ldh inhibition or depletion.

Other stem cells, as well as cancer cells, share their metabolic profile, enhanced glycolysis, with ESCs. For example, hair follicle stem cells (HFSCs) maintain a high capacity for glycolytic metabolism, and a loss of *Ldha* prevents the activation of dormant HFSCs [36]. Similarly, in muscle stem cells (MuSCs), both the knockout of *AMPKα1* and overexpression of LDH lead to higher glycolysis and self-renewal rates. The inhibition of LDH activity returns the self-renewal of *AMPKα1* KO MuSCs to a normal level [37]. All these data suggest that protein lactylation might have a broader implication in cell fate regulation, in addition to ESC self-renewal and XEN differentiation.

In summary, our results reveal that glycolysis-derived lactate and protein lactylation, particularly Esrrb lactylation, provides a novel mechanistic link between glycolysis and cell fate regulation.

## 4. Materials and Methods

### 4.1. Cell Culture and Transfection

ESCs were cultured on 0.2% gelatin-coated tissue culture plates in a medium containinf 85% Dulbecco’s Modified Eagle Medum (DMEM) (Gibco, Waltham, MA, USA) + 15% fetal bovine serum (FBS) (HyClone, Logan, UT, USA) + 2 mM L-glutamine (Invitrogen, Carlsbad, CA, USA) + 100 units/mL penicillin and 100 µg/mL streptomycin (Invitrogen) + 0.1 mM nonessential amino acids (Invitrogen) + 0.1 mM β-mercaptoethanol (Sigma-Aldrich, St. Louis, MO, USA) + 1000 units/mL LIF. The medium was changed every day, and ESCs were passaged every 2–3 days. All cell lines were cultured at 37 °C with 5% CO_2_.

XEN differentiation was performed as described elsewhere [38]. We plated 25K cells in a well of a 12-well plate in XEN medium. The XEN medium was prepared as follows: RPMI 1640 medium (Gibco) + 15% FBS + 2 mM L-glutamine + 100 units/mL penicillin and 100 µg/mL streptomycin + 0.1 mM β-mercaptoethanol + 10 ng/mL Activin A (PeproTech, Cranbury, NJ, USA) + 0.1 μM Retinoic acid (MedChemExpress, Shanghai, China).

Transfection was performed with Lipo8000™ (Beyotime, Shanghai, China) according to the manufacturer’s instructions. All ESC lines were tested for mycoplasma contamination every 2 weeks.

### 4.2. Plasmid Construction

pLentil-Cas9-puro was used to construct gene knockout plasmids targeting Esrrb. The sgRNA oligonucleotides were designed using the website https://www.zlab.bio/guide-design-resources (accessed on 23 January 2021). The sgRNA sequence targeting Esrrb was 5′-TGGCGTCCGACGAGCCGCTGGGG-3′. PB-CAG-3xFLAG was used to construct overexpression plasmids. Esrrb was amplified and cloned from complementary DNA (cDNA) of ESCs. The Esrrb coding region was inserted into PB-CAG-3xFLAG through seamless cloning (Beyotime). KR and KQ Esrrb mutants were constructed through PCR mutagenesis. The primers are listed in Table S1.

### 4.3. Quantitative Reverse-Transcription PCR (qRT-PCR)

The total RNA was extracted from cells using TRIZOL (Ambion, Austin, TX, USA) according to the manufacturer’s recommendations. Reverse transcription was performed to generate cDNA using the Reverse Transcription kit (GenStar, Beijing, China). We performed the PCR reaction using a RealStar Probe Fast Mixture kit (GenStar) and a Quantitative PCR machine (Bio-Rad, Hercules, CA, USA). The primers are listed in Table S1. 

### 4.4. Colony-Forming Assay and Alkaline Phosphatase (AP) Staining

A total of 500 cells were plated on a 12-well plate. After 6 days of culture, images were captured using an Olympus IX81 (Nishi-Shinjuku, Japan). The number of colonies was counted. The diameters of colonies were quantified with Image J 1.51 software (National Institutes of Health). ESCs were stained with an alkaline phosphatase substrate kit III (Vector Laboratories, Newark, CA, USA) according to the manufacturer’s instructions, and images were taken with a Nikon TS100 (Tokyo, Japan).

### 4.5. Western Blot

Cells were lysed with lysis buffer (a cocktail of 20 mM Tris-HCl pH 8.0, 60 mM NaCl, 0.2% glycerol, 0.02% NP-40, 0.04 mM EDTA, 1 mM PMSF, and 1.52 mg/mL protease inhibitor). Proteins were analyzed with SDS-PAGE gel and transferred onto a polyvinyl difluoride membrane (PVDF) (Millipore, Temecula, CA, USA). The PVDF membrane was incubated with primary antibodies overnight at 4 °C. The primary antibodies used were anti-Kla (PTM BIO, PTM-1401,1:1000), anti-β-Tubulin (Abmart, M20005H, 1:5000, Shanghai, China), anti-Ldha (Cell Signaling Technology, 2012S, 1:1000, Danvers, MA, USA), anti-Nanog (Bethyl Laboratories, A300-397A, 1:2000), anti-Oct4 (Santa Cruz Biotechnology, sc-5279, 1:1000, Dallas, TX, USA), anti-Esrrb (Perseus Proteomics, H6705-00, 1:1000, Tokyo, Japan), and anti-FLAG (Sigma-Aldrich, F1804, 1:3000). After washing with TBS 3 times, the membrane was incubated with HRP-conjugated secondary antibodies at room temperature for 2 h. The secondary antibodies used were donkey anti-Rabbit (Cytiva, NA934V, 1:5000, Marlborough, MA, USA), goat anti-Rabbit (Santa Cruz, sc-2354, 1:1000), and sheep anti-Mouse (Cytiva, NA931V, 1:5000, Marlborough, MA, USA). ECL solution (Shanghai Epizyme Biomedical Technology Co., Ltd., Shanghai, China) was added, and signals were detected using an automatic chemiluminescence imaging analysis system (Tanon, Shanghai, China).

### 4.6. Immunoprecipitation

Ten million cells were lysed in lysis buffer. Immunoprecipitation was carried out using anti-FLAG M2 Magnetic Beads or specific antibodies followed by precipitation with 10 μL of 50% protein A or G agarose beads (Cytiva) on a rotor overnight at 4 °C. After washing with lysis buffer three times, beads were re-suspended in 30 μL 2×SDS-PAGE Plus Sample Buffer (Genstar) and boiled for 5 min at 100 °C to elute. Then, the samples were resolved with SDS-PAGE gel and subjected to Western blot. 

### 4.7. Chromatin Immunoprecipitation-Seq (ChIP–Seq)

ChIP was carried out with the SimpleChIP Enzymatic Chromatin IP Kit (Cell Signaling Technology, 9003S) according to the manufacturer’s instructions. The antibodies for immunoprecipitation were anti-FLAG (Sigma-Aldrich, F1804, 1:300). Purified ChIP DNA was used for library construction following the Illumina ChIP–seq library generation protocol (New England Biolabs, Ipswich, UK). Sequencing was performed using Novogene (Tianjin, China). 

### 4.8. Statistical Analysis

At least 3 independent experiments were analyzed, and data are presented as average ± SD. Statistical analysis was performed with an unpaired two-tailed Student’s *t* test or two-way ANOVA, which are indicated in the figure legends. Statistically significant *p* values are indicated in the figures as follows: * *p* < 0.05; ** *p* < 0.01; *** *p* < 0.001.

## Data Availability

The sequencing data generated in this study have been deposited into the Gene Expression Omnibus database under accession number GSE237618 (access token: evkrmokufvgbrmb).

## Supplementary Materials

The supporting information can be downloaded at: https://www.mdpi.com/article/10.3390/ijms25052692/s1.
