# Supplementary material for: Glycolysis-Stimulated Esrrb Lactylation Promotes the Self-Renewal and Extraembryonic Endoderm Stem Cell Differentiation of Embryonic Stem Cells
Source: Int J Mol Sci. 2024 Feb 26;25(5):2692. doi: 10.3390/ijms25052692 (PMC10932464; doi:10.3390/ijms25052692)
Supplement: Supplementary file 1 [file ijms-25-02692-s001.zip › Supplemental data .pdf]

Glycolysis-stimulated Esrrb Lactylation Promotes the Self-renewal and XEN Differentiation of Embryonic Stem Cells

Qiman Dong[1], Qingye Zhang[1], Xiaoqiong Yang[1], Shanshan Nai[1], Xiaoling Du[1], Lingyi Chen[1,*]

[1] Institute of Translational Medicine, Tianjin Union Medical Center, State Key Laboratory of Medicinal Chemical Biology, Tianjin Key Laboratory of Protein Sciences, Frontiers Science Center for Cell Responses, National Demonstration Center for Experimental Biology Education and College of Life Sciences, Nankai University, Tianjin, China

* Corresponding author: Lingyi Chen

Email: lingyichen@nankai.edu.cn

Keywords: Lactylation, Esrrb, Self-renewal, XEN differentiation, Embryonic stem cells.

Figure supplement

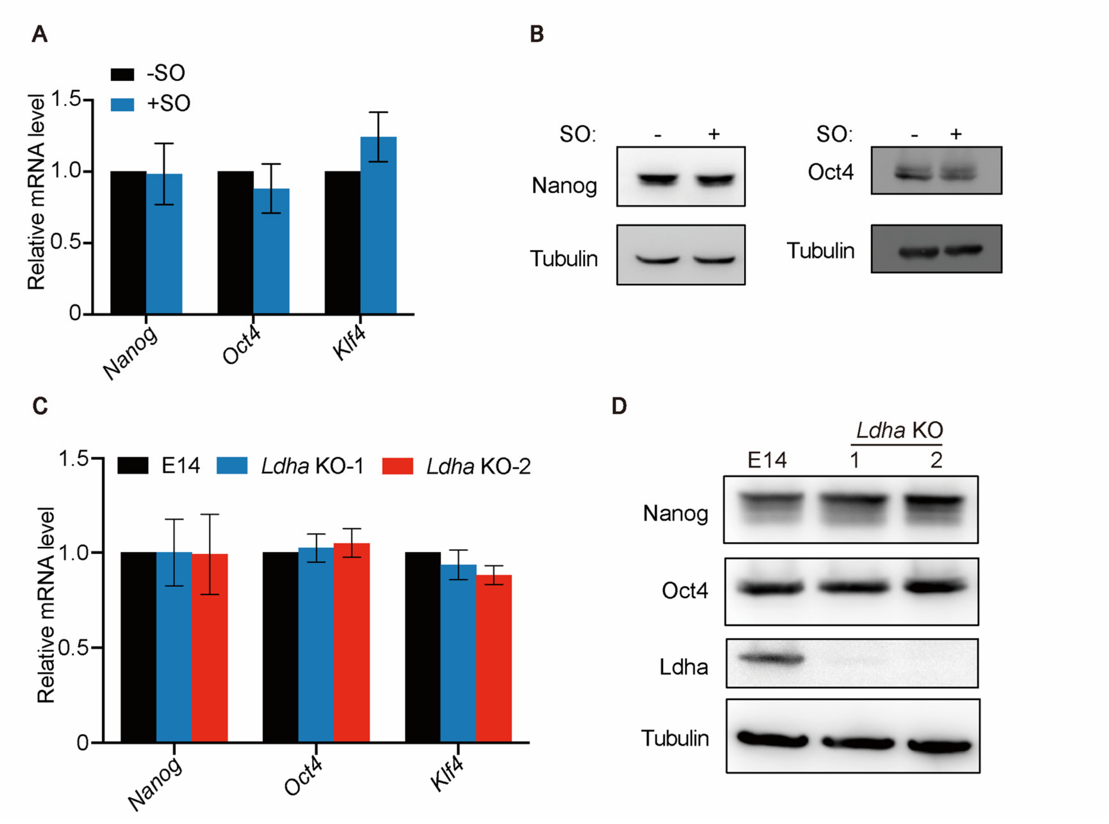

Figure S1. Inhibition of Ldha does not affect the expression of pluripotency genes.

A, B E14 ESCs were treated with and without 20 mM SO for 24 h. The cells were harvested for qRT-PCR (**A**) and Western blot (**B**). **A** The expression levels of pluripotent genes, *Nanog*, *Oct4* and *Klf4*, were examined. **B** The expression levels of Nanog and Oct4 were examined. **C** The expression of pluripotent genes, *Nanog*, *Oct4* and *Klf4*, in E14 and *Ldha* KO ESCs, detected by qRT-PCR. **D** The expression of Nanog and Oct4 in E14 and *Ldha* KO ESCs, detected by Western blot. For qRT-PCR, n=3. Data are presented as average ± SD. Statistical analysis was performed with unpaired two-tailed Student's t test.

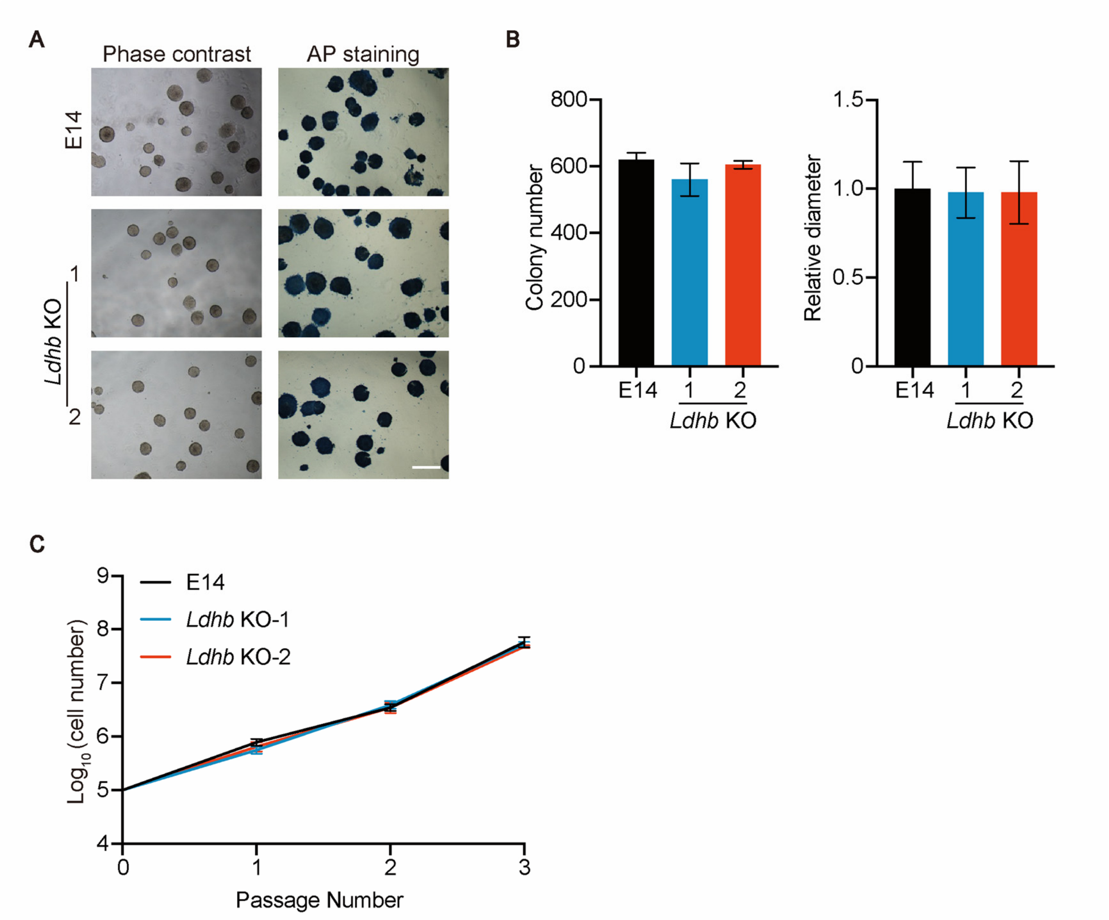

Figure S2. *Ldhb* knockout does not affect the self-renewal of ESCs. A Colony forming assay of E14 and *Ldhb* KO ESCs. Representative images of colony forming assay and AP staining are shown. Scale bar: 500 μm. **B** The number and diameter of E14 and *Ldhb* KO ESC colonies described in (**A**). **C** Growth curves of E14 and *Ldhb* KO ESCs. For growth curves and colony formation, n=3. Data are presented as average ± SD. Statistical analysis was performed with unpaired two-tailed Student's t test.

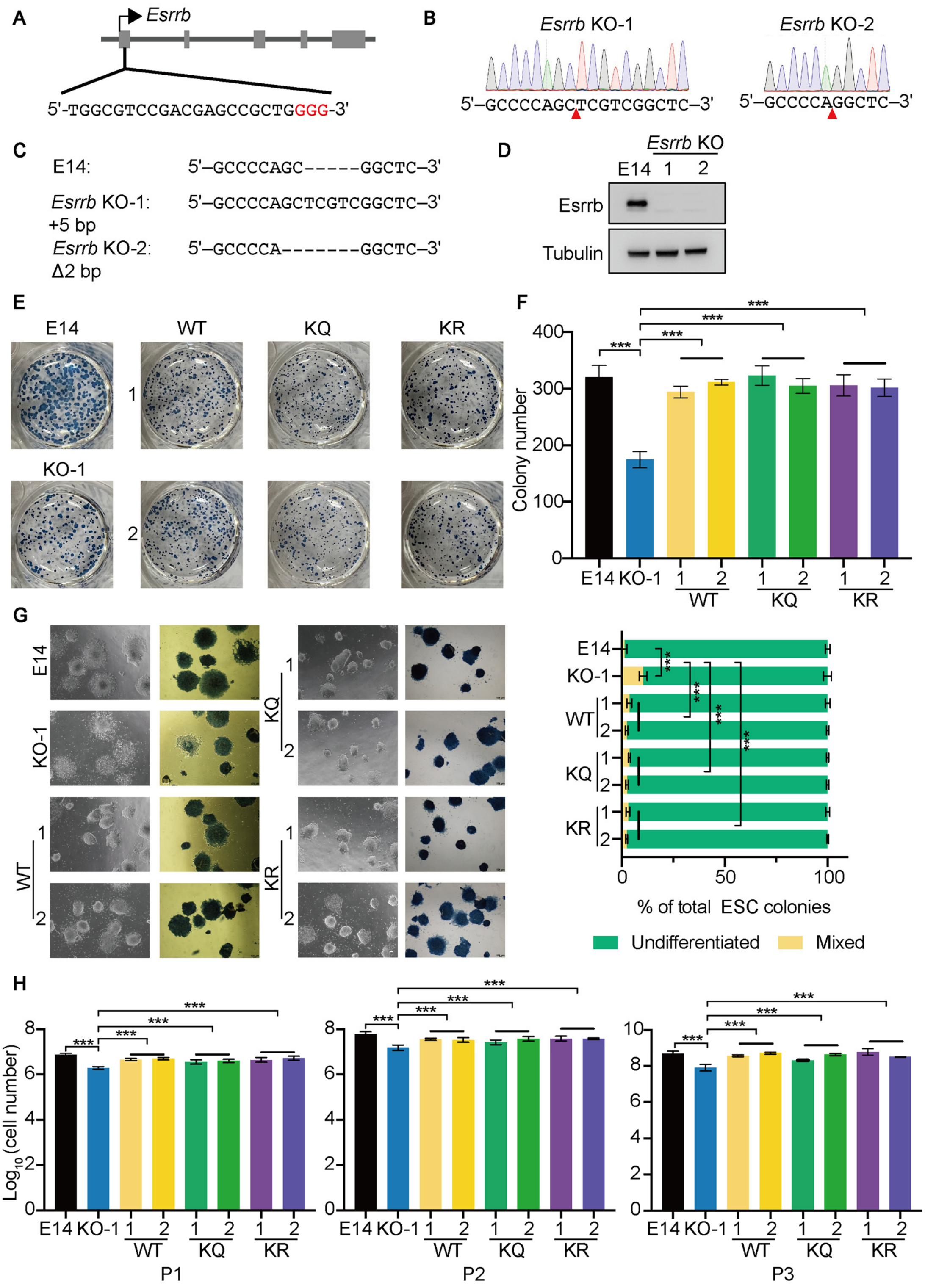

Figure S3. WT, KQ, and KR Esrrb show no difference in pluripotency maintenance under optimal condition. A A schematic illustration of the experimental design to knock out *Esrrb*. Gray rectangles represent the exons of *Esrrb*. The sgRNA

targeting sequence is shown, and the PAM motif is marked in red. **B** Sequencing chromatograph of the *Esrrb* gene around the Cas9 targeting site in *Esrrb* KO-1 and KO-2 ESCs. Red triangles mark the indel mutations. **C** Sequence alignment of the *Esrrb* gene around the Cas9 targeting site in E14 and *Esrrb* KO ESCs. **D** The expression of *Esrrb* in E14 and *Esrrb* KO ESCs, detected by Western blot. **E** Colony forming assay of E14, *Esrrb* KO-1, WT, KQ, and KR ESCs cultured in ESC medium. **F** The number of ESC colonies described in (**E**). **G** The bright field images of colony-forming assays (**E**), before and after AP staining, are shown in the left. The right panel showed the fractions of undifferentiated, mixed, and differentiated colonies in each cell line. **H** The proliferation of E14, *Esrrb* KO-1, WT, KQ, and KR ESCs. ESCs were plated and cultured for 3 passages. Cell numbers were counted at each passage and plotted. Three independent experiments were analyzed, and data are presented as average ± SD. Statistical analysis was performed with two-way ANOVA. ***, $p < 0.001$. Scale bar: 100 μm.

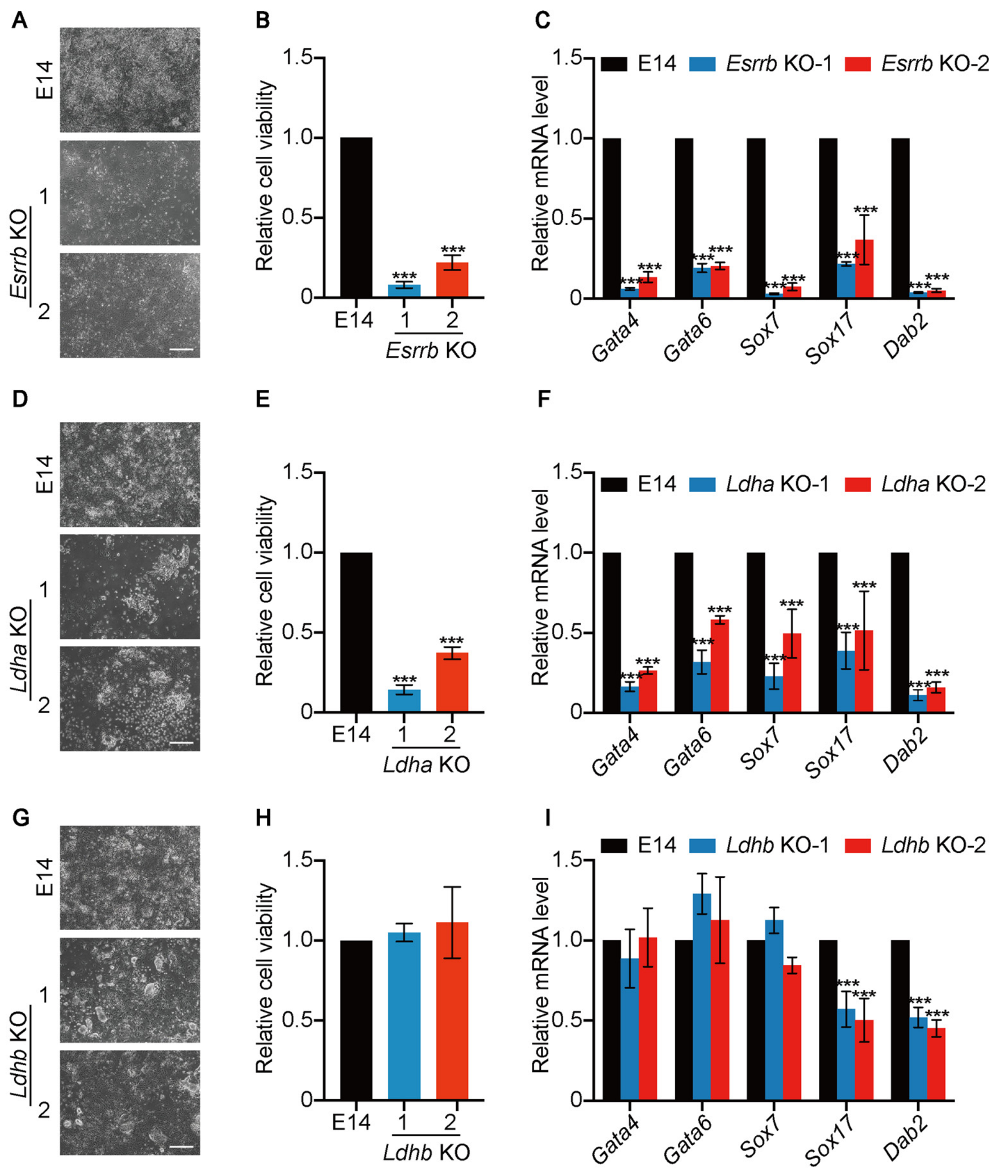

Figure S4. Knockout of *Esrrb* and *Ldha*, but not *Ldhb*, impairs XEN differentiation. A The representative phase-contrast images of XEN cells differentiated from E14, *Esrrb* KO ESCs. **B** The relative cell viability of XEN cells described in (**A**). **C** The expression of selected XEN genes in XEN cells described in (**A**). **D** The representative phase-contrast images of XEN cells differentiated from E14, *Ldha* KO ESCs. **E** The relative cell viability of XEN cells described in (**D**). **F** The expression of selected XEN genes in XEN cells described in (**D**). **G** The

representative phase-contrast images of XEN cells differentiated from E14, *Ldhb* KO ESCs. **H** The relative cell viability of XEN cells described in (**G**). **I** The expression of selected XEN genes in XEN cells described in (**G**). Three independent experiments were analyzed, and data are presented as average ± SD. Statistical analysis was performed with unpaired two-tailed Student's t test. ***, $p < 0.001$. Scale bar: 500 μm.

Supplemental Tables

Table S1. Primers used in this study

		Forward	Reverse
quantitative RT-PCR primers	*Nanog*	TACAAGGGTCTGCTACTGAGATGC	TTGGGACTGGTAGAAGAATCAGGG
	Oct4	ATCAGCTTGGGCTAGAGAAGGATG	AAAGGTGTCCCTGTAGCCTCATAC
	Klf4	AGCCACCCACACTTGTGACTAT	AGTGGTAAGGTTTCTCGCCTGT
	β-Actin	CAGAAGGAGATTACTGCTCTGGCT	TACTCCTGCTTGCTGATCCACATC
	Tbx3	TTCACAACTCTCGGTGGATGGT	CGCTTGGGAAGGCCAAAGTAAA
	Gata4	GCTATGCATCTCCTGTCACTCAGA	CCAAGTCCGAGCAGGAATTTGAAG
	Gata6	CTTCTCCTTCTACACAAGCGACCA	ATACTTGAGGTCACTGTTCTCGGG
	Sox7	GCTCCTGCTTTTGGTGTAGC	GTCCTTGGGCAGTCATTCAT
	Sox17	GAGGGCCAGAAGCAGTGTTA	AGTGATTGTGGGGAGCAAGT
	Dab2	TCTCAGCCTGCATCTTCTGA	GAGCGAGGACAGAGGTCAAC
Mutagenesis primers	K228R	CGATTTCCCCACCTGCTAAAAGGCCATTGACTAAGA	TCTTAGTCAATGGCCTTTTAGCAGGTGGGGAAATCG
	K228Q	TCGAGACGATCTGAGTCAATGGCTTTTTAGCAGGTG	CACCTGCTAAAAAGCCATTGACTCAGATCGTCTCGA
	K232R	CTAGTAGATTCGAGACGATCCTAGTCAATGGCTTTTTAGCA	TGCTAAAAAGCCATTGACTAGGATCGTCTCGAATCTACTAG
	K232Q	CTTAGTCAATGGCTGTTTAGCAGGTGGGGAAATCG	CGATTTCCCCACCTGCTAAACAGCCATTGACTAAG
ChIP-qPCR primers	*Nanog*	TCGCCAGGGTCTGGAGGTGC	TCCCACCTGCAGGGTCCACC
	Tbx3	CGGTCTAGGTTGTCCTGTAGTA	CCATGCATAGGTGGATCTTAGG
	Intergenic-Chr.8	AAGGGGCCTCTGCTTAAAA	AGAGCTCCATGGCAGGTAGA
